# Microarray data on the comparison of transcript expression between normal and *Pt-Delta* RNAi embryos in the common house spider *Parasteatoda tepidariorum*

**DOI:** 10.1016/j.dib.2019.104350

**Published:** 2019-08-05

**Authors:** Hiroki Oda, Yasuko Akiyama-Oda

**Affiliations:** a Laboratory of Evolutionary Cell and Developmental Biology, JT Biohistory Research Hall, 1-1 Murasaki-cho, Takatsuki, Osaka 569-1125, Japan; b Laboratory of Biohistory, Department of Biological Sciences, Graduate School of Science, Osaka University, Japan; c Microbiology and Infection Control, Osaka Medical College, Takatsuki, Osaka, Japan

**Keywords:** Arthropod, *Parasteatoda tepidariorum*, Embryogenesis, Pattern formation, Gene expression, Notch signaling

## Abstract

We conducted a custom microarray experiment to detect the differences in the transcript expression levels between untreated (normal) and *Pt-Delta*-RNAi embryos at late stage 6 in the common house spider *Parasteatoda tepidariorum*. The array probes were designed based on accumulated EST and cDNA sequences. The microarray dataset has been deposited in the Gene Expression Omnibus (GEO) Database at the National Center for Biotechnology Information (NCBI) under the accession GSE113064. The expression of the transcripts selected based on the detected differences was examined in embryos by whole-mount *in situ* hybridization.

**Table:** Specifications Table

Subject area	Biology
More specific subject area	Developmental Biology
Type of data	Tab-delimited text, table, image
How data was acquired	Custom oligonucleotide microarray, whole-mount *in situ* hybridization
Data format	Processed values and raw images
Experimental factors	No biological or technical replicates
Experimental features	Total RNA was extracted from *Pt-Delta* parental RNAi and untreated embryos at late stage 6
Data source location	Osaka, Japan
Data accessibility	The microarray dataset has been deposited in the Gene Expression Omnibus database at NCBI under the accession GSE113064. https://www.ncbi.nlm.nih.gov/geo/query/acc.cgi?acc= GSE113064 The image data have been deposited in the Mendeley Data repository. https://doi.org/10.17632/r79vg2ctr2.3.

**Table:** 

**Value of the data**
• The dataset is useful for identifying the candidate genes whose expression is regulated by Delta-Notch signaling in *P. tepidariorum* embryos.
• The dataset is useful for identifying the genes whose expression marks specific cell types or regions of *P. tepidariorum* embryos.
• The dataset is useful for investigating the gene regulatory networks in the embryonic development of spider.

## 1 Data

**Fig. 1:**
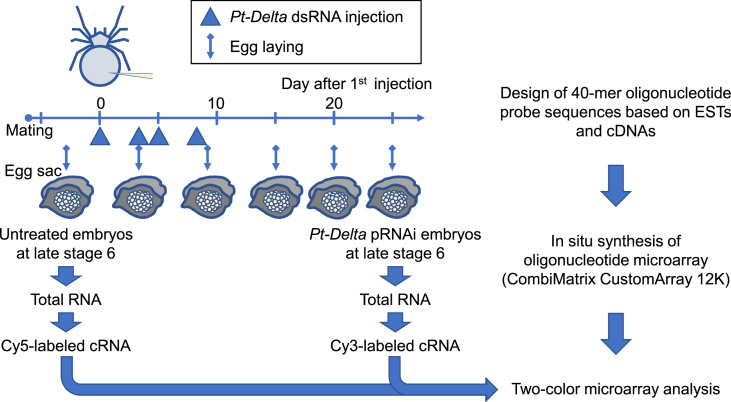
Flowchart of the microarray experiment.

**Fig. 2:**
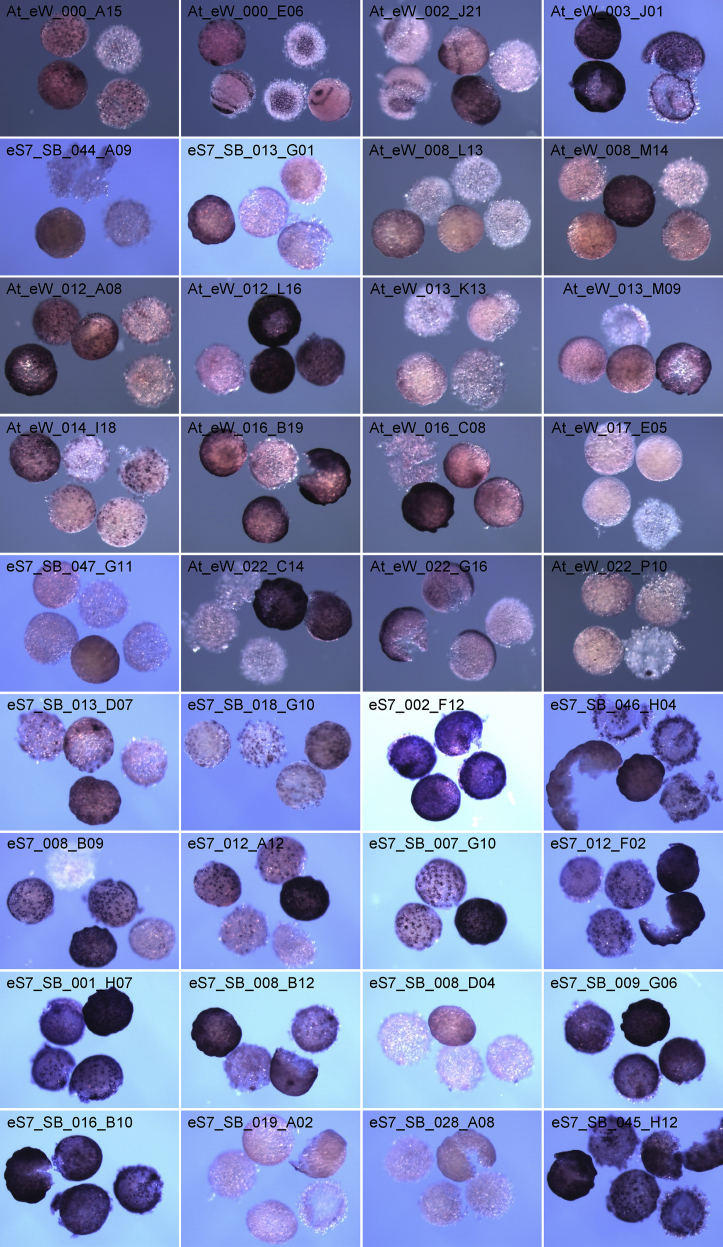
Staining of stage 5–8 embryos for selected transcripts by WISH.

Transcript expression was compared between untreated (normal) and *Pt-Delta* RNAi-treated (*Pt-Delta* RNAi) embryos at late stage 6 using a Combimatrix custom microarray in 12K format (Fig. 1), which was designed based on the accumulated *Parasteatoda tepidariorum* EST and cDNA sequences. The microarray dataset deposited in the GEO Database at NCBI (GSE113064) consists of a data table showing the details of probe sequences for array spots (Platform: GPL24882) and that showing the ratio of [*Pt-Delta* RNAi]/[normal] for each array spot (Sample: GSM3095654). Values of the [*Pt-Delta* RNAi]/[normal] ratio from control probes are shown in Table 1. EST clones that showed the ratio of [*Pt-Delta* RNAi]/[normal] of <0.6 for at least one array probe are listed together with their details in Table 2. Whole-mount *in situ* hybridizations (WISHs) of stage 5–8 embryos showing expression of the transcripts related to these EST clones are displayed in Fig. 2. The original images, including high-magnification images showing the transcript expression patterns and nuclear stains, are available in a data repository [1].

**Table 1:** Values of the [*Pt-Delta* RNAi]/[normal] ratio from control probes in the microarray analysis.

EST/cDNA clone	Gene product	Spot position numbers in MA^a^	Sequence accession	AUGUSTUS gene model^b^	NCBI GeneID	Ratio (mean ± s.d.)^c^
At_eW_003_D02	*alpha-catenin*	1719/4149/8989/11508	AB433907	g13984	LOC107439705	0.880 ± 0.039
At_eW_003_D02	*alpha-catenin*	623/6036/6733/11968	AB433907	g13984	LOC107439705	0.950 ± 0.265
eS7_003_G08	*elongation factor 1-alpha*	1697/2124/4972	AB433908	g27264	LOC107441347	1.163 ± 0.040
eS7_003_G08	*elongation factor 1-alpha*	6130/9614/11011	AB433908	g27264	LOC107441347	1.061 ± 0.072
eS7_SB_037_C01	*histone H3*	565/1580/1610/4874	AB433909	g1955	LOC107447866	0.965 ± 0.074
eS7_SB_037_C01	*histone H3*	946/6236/6382/9005	AB433909	g1955	LOC107447866	0.834 ± 0.085
At_O091	*Delta*	3003/6080/9491/10203	AB287420	g25248	LOC107456525	1.022 ± 0.099
At_O091	*Delta*	3364/10373/10432/12130	AB287420	g25248	LOC107456525	1.164 ± 0.212
At_O034	*caudal*	4150/6419/9162/12485	AB096075	g12643	LOC107437910	0.379 ± 0.068
At_O034	*caudal*	6936/8225/8858/10344	AB096075	g12643	LOC107437910	0.358 ± 0.026
At_O035	*caudal*	3904/4818/8215/10264	AB096075	g12643	LOC107437910	0.150 ± 0.009
At_O045	*twist*	6351/7485/7754/9334	AB167807	g14287	LOC107440133	1.464 ± 0.098
At_O045	*twist*	1481/3243/7334/10872	AB167807	g14287	LOC107440133	1.058 ± 0.074
At_O046	*twist*	2538/3783/7509/8625	AB167807	g14287	LOC107440133	1.070 ± 0.095
At_O029	*hedgehog*	3925/5404/11012/11143	AB125742	g4322	LOC107451809	0.436 ± 0.037
At_O029	*hedgehog*	432/865/4215/4950	AB125742	g4322	LOC107451809	0.623 ± 0.102
At_O030	*hedgehog*	2406/4772/4944/7704	AB125742	g4322	LOC107451809	0.905 ± 0.053
At_O032	*orthodenticle*	1941/5594/10660/11559	AB096074	g9172	LOC107457189	0.298 ± 0.040
At_O032	*orthodenticle*	838/3555/8545/9265	AB096074	g9172	LOC107457189	0.878 ± 0.155
At_O071	*odd-paired*	6865/9525/9551/11052	AB605264	g12202	LOC107437305	0.716 ± 0.023
At_O071	*odd-paired*	347/3228/4356/10492	AB605264	g12202	LOC107437305	1.021 ± 0.161

a Two or three 40-mer oligonucleotide sequences were designed from each EST/cDNA sequence for the microarray (MA). The spot position numbers in MA link the data in this report and those deposited in the GEO database.

b AUGUSTUS gene models (aug3) were described by Schwager et al. (2017) [6].

c The average value based on four or three spot replicates in a MA.

**Table 2:** List of EST clones selected based on the [*Pt-Delta* RNAi]/[normal] ratio (<0.6) in the microarray analysis.

EST clone	Spot position number in MA^a^	Sequence accession	AUGUSTUS gene model^b^	NCBI GeneID	Ratio	WISH probe^c^	Exp.^d^
At_eW_000_A15	10599	FY216311	g9542	LOC107437620	0.597	At_eW_000_A15*	end
eS7_SB_035_H06	7990	FY380468	g9542	LOC107437620	0.550	eS7_SB_035_H06	
eS7_SB_035_H06	12394	FY380468	g9542	LOC107437620	0.556	eS7_SB_035_H06	
At_eW_000_E06	8245	FY216397	g15506	LOC107449884	0.453	At_eW_000_E06*	ect (ptn)
At_eW_000_J22	11227	FY216533	g15506	LOC107449884	0.506	At_eW_000_J22	ect (ptn)
At_eW_000_J22	9441	FY216533	g15506	LOC107449884	0.519	At_eW_000_J22	ect (ptn)
At_eW_002_J21	2951	FY217255	g6063	LOC107454132	0.554	At_eW_002_J21*	ect (ptn)
At_eW_002_J21	4632	FY217255	g6063	LOC107454132	0.508	At_eW_002_J21*	ect (ptn)
At_eW_003_J01	4517	FY217568	g12522	LOC110282483	0.592	At_eW_003_J01*	mes
At_eW_005_P09	6960	FY218402	g12522	LOC110282483	0.581	eS7_SB_032_G07	mes
At_eW_004_F14	12040	FY217823	g27319	LOC107441543	0.559	eS7_SB_044_A09*	
At_eW_007_I04	12305	FY218925	g18068	LOC107444748	0.494	eS7_SB_013_G01*	
At_eW_007_I04	6439	FY218925	g18068	LOC107444748	0.582	eS7_SB_013_G01*	
At_eW_008_L13	11956	FY219308	g16765	n/a	0.591	At_eW_008_L13*	
At_eW_008_M14	7886	FY219331	g19785	LOC107447481	0.516	At_eW_008_M14*	
At_eW_012_A08	3184	FY220282	g15726	LOC107451427	0.574	At_eW_012_A08*	end + ex
At_eW_012_L16	11579	FY220479	g3986	LOC107451377	0.588	At_eW_012_L16*	
At_eW_013_K13	9406	FY220732	g16422	n/a	0.599	At_eW_013_K13*	
At_eW_013_M09	5434	FY220762	g13957	LOC107439672	0.535	At_eW_013_M09*	
At_eW_014_I18	2396	FY220952	g27732	LOC107442734	0.599	At_eW_014_I18*	end
At_eW_016_P19	9236	FY221580	g27732	LOC107442734	0.576	At_eW_016_P19	end
eS7_SB_012_D04	8294	FY378611	g27733	LOC107442734	0.524	eS7_SB_012_D04	end
eS7_SB_012_D04	6032	FY378611	g27733	LOC107442734	0.567	eS7_SB_012_D04	end
At_eW_016_B19	4625	FY221307	g1624	LOC107447145	0.498	At_eW_016_B19*	
At_eW_016_C08	10970	FY221318	g17631	n/a	0.562	At_eW_016_C08*	
At_eW_017_E05	11338	FY221647	g7733	LOC107447731	0.589	At_eW_017_E05*	
At_eW_021_K03	11455	FY223147	g25961	LOC107436162	0.577	eS7_SB_047_G11*	
At_eW_022_C14	7174	FY223344	g58	LOC107440897	0.552	At_eW_022_C14*	
At_eW_022_C14	5932	FY223344	g58	LOC107440897	0.483	At_eW_022_C14*	
At_eW_022_G16	10754	FY223437	g11061	LOC107457313	0.569	At_eW_022_G16*	ect (ptn)
At_eW_022_P10	11105	FY223640	g26989	LOC107440147	0.474	At_eW_022_P10*	cm
At_eW_025_H20	7926	FY224563	g23736	LOC107453117	0.572	At_eW_025_H20	
eS7_SB_013_D07	10632	FY378703	g23736	LOC107453117	0.551	eS7_SB_013_D07*	end
eS7_001_G09	2121	FY376646	g2117	LOC107448180	0.598	eS7_SB_018_G10*	end
eS7_002_F12	5636	FY376729	g2859	LOC107449611	0.588	eS7_002_F12*	
eS7_006_B01	7269	FY377014	g15364	LOC107448906	0.598	eS7_SB_046_H04*	end
eS7_008_B09	6413	FY377193	g7446	LOC107444045	0.597	eS7_008_B09*	end
eS7_012_A12	11123	FY377551	g3860	LOC107451189	0.570	eS7_012_A12*	end
eS7_012_C04	6762	FY377567	g9240	LOC107445106	0.512	eS7_SB_007_G10*	end
eS7_012_F02	12018	FY377596	g18588	LOC107445478	0.574	eS7_012_F02*	end
eS7_SB_001_H07	633	FY377714	g8636	LOC107456533	0.579	eS7_SB_001_H07*	end + ex
eS7_SB_008_B12	11074	FY378225	g15926	LOC107441456	0.553	eS7_SB_008_B12*	cm
eS7_SB_008_D04	9585	FY378241	g11817	LOC107436785	0.524	eS7_SB_008_D04*	
eS7_SB_009_G06	11640	FY378373	g16253	LOC107441912	0.595	eS7_SB_009_G06*	
eS7_SB_016_B10	12337	FY378949	g18790	LOC107445841	0.565	eS7_SB_016_B10*	end
eS7_SB_016_B10	7166	FY378949	g18790	LOC107445841	0.535	eS7_SB_016_B10*	end
eS7_SB_019_A02	12357	FY379131	g8457	LOC107456289	0.493	eS7_SB_019_A02*	
eS7_SB_028_A08	8536	FY379919	g4630	LOC107452244	0.574	eS7_SB_028_A08*	
eS7_SB_045_H12	11661	FY381390	g25109	LOC107455614	0.536	eS7_SB_045_H12*	end

n/a, not applicable.

a The spot position numbers in the microarray (MA) link the data in this report and those deposited in the GEO database.

b AUGUSTUS gene models (aug3) were described by Schwager et al. (2017) [6].

c EST clone used for the synthesis of RNA probes for whole-mount *in situ* hybridization (WISH). In some cases, a different EST clone including the MA probe sequence was used for WISH. The WISH data from EST clones indicated by asterisks are displayed in Fig. 1.

d Expression in specific cell types (end, endoderm; ex, extraembryonic tissue; mes, mesoderm; ect, ectoderm; cm, cumulus mesenchymal cells) and/or specific patterns (ptn, patterned) as revealed by WISH.

## 2 Experimental design, materials and methods

The primary objective of this experiment was to identify the genes whose expression might be affected by parental RNA interference (pRNAi) against *Pt-Delta* in *P. tepidariorum* embryos [2]. Flow of the microarray experiment is schematically shown in Fig. 1.

### 2.1 Custom microarray design

40-mer oligonucleotide probes were designed based on the accumulated *P. tepidariorum* EST and cDNA sequences [2], [3] using OligoArray 2.1 [4] and embedded in a custom microarray (CombiMatrix CustomArray 12K, CustomArray, Inc.). There were single or multiple probes designed from each EST or cDNA sequence. Four or three spot replicates of control probes (Table 1) were included to validate the experiment. The details of the microarray design, including the probe sequences, are available from the GEO database (GPL24882).

### 2.2 Microarray analysis

A mated female was injected with approximately 1.5 μl of *Pt-Delta* dsRNA solution (2 μg/μl) 4 times at 2–3 days intervals. Embryos derived from an egg sac produced by the female one day before (normal) and 25 days after (*Pt-Delta* RNAi) the first injection of *Pt-Delta* dsRNA were used for RNA extraction. The total RNA was extracted from approximately 250 embryos at late stage 6 using MagExtractor (Toyobo). The RNA integrity was examined with an Agilent Bioanalyzer 2100. cRNA labeled with Cy3 or Cy5 was prepared from 2 μg of total RNA using RNA Transcript SureLABEL Core Kit (Takara). The cRNA probes were hybridized to microarray using Hybridization buffer (5X SSC, 0.1% SDS, 10% formamide) at 42 °C for 16–20 h. The microarray slide was scanned using a GenePix 4000B Scanner (Molecular Devices). There were no biological replicates. The obtained image was analyzed using an Array-Pro Analyzer ver. 4.5 (Media Cybernetics, Inc.). The quantitative data were subjected to Loess normalization. The ratio of the normalized intensity values ([*Pt-Delta* RNAi]/[normal]) for each probe was calculated. The probes for alpha-catenin (GB_ACC: AB433907; GI: LOC107439705), elongation factor 1-alpha (GB_ACC: AB433908; GI: LOC107441347), and histone H3 (GB_ACC: AB433909; GI, LOC107447866) served as negative controls, and the probes for a homolog of *Drosophila caudal*, *Pt-cad* (GB_ACC: AB096075; GI: LOC107437910) [2], served as positive controls to validate the experiment (Table 1).

### 2.3 Embryo staining

EST clones that were selected based on the [*Pt-Delta* RNAi]/[normal] ratio (<0.6) were used for the synthesis of Digoxigenin-labeled RNA probes for WISH. Normal embryos at stages 5–8 were stained by WISH as described [5]. They were counter-stained with 4',6-diamidino-2-phenylindole for visualization of the nuclei. The stained embryos were photographed using a stereomicroscope (SZX12, Olympus) equipped with a color CCD camera (C7780-10, Hamamatsu Photonics) and examined using a fluorescence microscope (Axiophot 2, Zeiss).
